# Sexual, bladder and bowel function following different minimally invasive techniques of radical hysterectomy in patients with early-stage cervical cancer

**DOI:** 10.1007/s12094-021-02632-7

**Published:** 2021-05-18

**Authors:** K. Baessler, S. Windemut, V. Chiantera, C. Köhler, J. Sehouli

**Affiliations:** 1 Department of Gynecology with Center for Oncological Surgery, Charité-Universitätsmedizin Berlin, Berlin, Germany; 2 Pelvic Floor Centre Franziskus and St Joseph Hospital Berlin, Budapester Str. 15-19, 10787 Berlin, Germany; 3 Department of Gynecology, Vivantes Hospital Am Urban, Berlin, Germany; 4 Department of Gynecologic Oncology, University of Palermo, Palermo, Sicilia Italy; 5 Department of Gynecology, Medical Faculty, University of Cologne, Cologne, Germany

**Keywords:** Pelvic floor function, Cervical cancer, Quality of life, Minimally invasive surgery, Urinary incontinence, Sexual function

## Abstract

**Purpose:**

Despite the establishment of radical surgery for therapy of cervical cancer, data on quality of life and patient-reported outcomes are scarce. The aim of this retrospective cohort study was to evaluate bladder, bowel and sexual function in women who underwent minimally invasive surgery for early-stage cervical cancer.

**Methods:**

From 2007–2013, 261 women underwent laparoscopically assisted radical vaginal hysterectomy (LARVH = 45), vaginally assisted laparoscopic or robotic radical hysterectomy (VALRRH = 61) or laparoscopic total mesometrial resection (TMMR = 25) and 131 of them completed the validated German version of the Australian Pelvic Floor Questionnaire (PFQ). Results were compared with controls recruited from gynecological clinics (*n* = 24) and with urogynecological patients (*n* = 63).

**Results:**

Groups were similar regarding age, BMI and parity. The TMMR group had significantly shorter median follow-up (16 months versus 70 and 36 months). Postoperatively, deterioration of bladder function was reported by 70%, 57% and 44% in the LARVH, VARRVH and TMMR groups, respectively (*p* = 0.734). Bowel function was significantly worse after TMMR with a higher deterioration rate in 72 versus 43% (LARVH) and 47% (VARRVH) with a correspondingly higher bowel dysfunction score of 2.9 versus 1.5 and 1.8, respectively and 1.8 in urogynaecological patients. Sexual dysfunction was common in all surgical groups. 38% considered their vagina too short which was significantly associated with deep dyspareunia.

Compared with controls, surgical groups had significantly increased PFQ scores.

**Conclusion:**

Pelvic floor dysfunction commonly deteriorates and negatively impacts on quality of life after minimally invasive radical hysterectomy, especially bowel function after TMMR. Pelvic floor symptoms should routinely be addressed pre- and postoperatively.

## Introduction

Cervical cancer is one of the most frequent and challenging diseases worldwide [1]. Besides oncological aspects, quality of life like pelvic floor function including sexuality is relevant but underreported in scientific reports [2, 3]. Surgery remains the cornerstone in the treatment of early cervical cancer. Abdominal radical hysterectomy has dominated surgical techniques for decades with complications like persisting voiding problems in up to 41% [4], bowel symptoms in up to 58% [5], sexual dysfunction in up to 60% [4, 6] and lymphedema in up to 19% [7, 8] of cases. Some studies comparing open and laparoscopic radical hysterectomy demonstrated similar oncological outcome [2, 9] whereas a recent randomized controlled trial (LACC trial) did not confirm this.

Several different minimally invasive techniques incorporating varying degrees of nerve-sparing have been described. During laparoscopically assisted radical vaginal hysterectomy (LARVH), the vaginal parametrial resection is preceded by laparoscopic staging and lymph node resection [10, 11]. Due to a long learning curve [11] and increased rates of urological complications during the vaginal part of the operation [12], an alternative technique was developed including creation of a vaginal cuff enclosing the cervix, laparoscopic radical hysterectomy with enbloc removal of uterus and parametria vaginally (vaginally assisted laparoscopic radical hysterectomy = VALRH) [13]. The laparoscopic part can also be performed Roboter-assisted (vaginally assisted robotic radical hysterectomy = VARRH) and achieved similar oncologic results [14].

The concept of abdominal total mesometrial resection (TMMR) is based on ontogenetic anatomy: The Müllerian compartment is completely removed during surgery apart from the vagina to maintain sexual function [15]. TMMR can also safely be performed laparoscopically [16].

Besides oncological safety, quality of life and pelvic floor function is important, especially in younger women. A recent meta-analysis described favorable bladder function after laparoscopic nerve-sparing radical hysterectomy [2]. Unfortunately, although eight studies reported bladder and bowel function, only two studies looked at sexual function.

The aim of this study was to evaluate bladder, bowel and sexual function in women who underwent LARVH, VALRH, VARRH or laparoscopic TMMR for early-stage cervical cancer. This is an ancillary report to Lucidi et al. [17] considering all women who answered a validated pelvic floor questionnaire at one center and comparing them to urogynecological patients and gynecologic controls.

## Methods

This retrospective cohort study was approved by the Charité Institutional Ethical Review Board. Written informed consent was obtained. All 261 women who underwent minimally invasive surgery for early-stage cervical cancer (≤ FIGO stage II) from 2005–2013, older than 18 years of age were asked to complete a validated pelvic floor questionnaire [18, 19]. From 2005–2007, LARVH was performed (*n* = 98), thereafter VALRH (*n* = 104) and VARRH (*n* = 24). Laparoscopic TMMR was performed from 2011 (*n* = 35).

Demographic and perioperative data were obtained from hospital charts. Postoperative voiding dysfunction was defined as postvoid residual of more than 50 ml when discharged. We noted first postoperative defecation and required doses of laxatives.

The German version of the Australian Pelvic Floor Questionnaire (PFQ) [20] assesses bladder, bowel, prolapse and sexual symptoms [18] and includes a validated post-treatment module evaluating the impression of improvement or deterioration in each pelvic floor domain [19]. We added particularly interesting questions: do you think your vagina is too short? (yes–no); has your ability to achieve orgasm changed after the operation? (unchanged, improved, worsened); have you been diagnosed with lymphedema? (yes–no).

Given the reported minimal important difference and effect sizes of the Australian PFQ and its German version [19, 21], we considered differences in domain scores of ≥1 as clinically important. The German validation paper [18] reported a global dysfunction score of 2.6 in healthy controls. To demonstrate a difference of 1.5 in the global score, 80% power and alpha = 0.05, 24 women had to be included in each group. However, we chose to invite all consecutive women to allow for multiple comparisons. To be able to judge the magnitude of pelvic floor dysfunction after surgery, we compared questionnaire scores with the control group recruited from gynecological clinics with non-malignant diseases (e.g., fibroids, *n* = 24) and women seeking care in our urogynecology unit (*n* = 66). None of these women were on anticholinergics or pessary treatment at the time of recruitment and none had undergone pelvic surgery including hysterectomy.

Statistical analysis was performed using IBM SPSS Statistics. For normally distributed variables, ANOVA was used with post hoc Bonferoni testing. For dichotomous variables, the Chi-square test or Fisher’s exact tests as appropriate were performed. For ordinal variables, the non-parametric Kruskal–Wallis test was employed.

In accordance with the journal’s guidelines, we will provide our data for the reproducibility of this study in other centers if such is requested.

## Results

Perioperative characteristics of all 261 women are given in Table 1. Operating time correlated negatively with age (*r* = − 0.144; *p* = 0.02) and positively with BMI (*r* = 0.186; *p* < 0.001). In the TMMR group the number of removed lymph nodes was significantly smaller compared to all other groups (*p* < 0.001). Radicality and perioperative complications including urinary tract infections, thrombosis and infection did not differ between operating techniques.

**Table 1:** Demographic and perioperative data, PIVER classification, TNM-stage and histopathological tumor type in all 261 women who underwent surgery for early-stage cervical cancer (LARVH-laparoscopically assisted radical vaginal hysterectomy, VALRH-vaginally assisted laparoscopic radical hysterectomy, TMMR- laparoscopic total mesometrial resection)

	LARVH *N* = 98	VALRH *N* = 104	Robotic VALRH *N* = 24	TMMR *N* = 35	*p*
Age (mean ± SD)	44.6 ± 11.5	46.3 ± 11.4	45.4 ± 9.1	47.8 ± 10.8	0. 456^a^
BMI (mean ± SD)	24.1 ± 4.3	25.2 ± 6.0	24.1 ± 3.6	24.3 ± 3.7	0*.*884^a^
Operating time (mean min ± SD)	318.2 ± 73.0	278.3 ± 82.1	336.7 ± 66.2	228.0 ± 54.1	< 0.001^a^
Number of lymph nodes (mean ± SD)	28.5 ± 14.0	28.4 ± 16.8	44.1 ± 15.0	15.6 ± 11.8	< 0.001^a^
PIVER					
II	60 (61%)	64 (62%)	20 (83%)	20 (57%)	0.153^b^
III	38 (39%)	40 (38%)	4 (17%)	15 (43%)	
TNM					
IA1L1	7 (7%)	11 (11%)	0	2 (6%)	0.430^b^
IA2	8 (8%)	4 (4%)	1 (4%)	3 (9%)	
IB1	74 (75%)	73 (70%)	23 (96%)	27 (77%)	
IB2	2 (2%)	9 (9%)	0	1 (3%)	
IIA1	1 (1%)	0	0	0	
IIA2	1 (1%)	0	0	0	
IIB	5 (5%)	7 (7%)	0	2 (6%)	
Hospital stay (mean ± SD)	12 ± 3	10 ± 3	10 ± 3	8 ± 2	< 0.001^a^

Percentages may not sum to 100 due to rounding

^a^ANOVA

^b^Chi-square test

At the time of hospital discharge, only 4/35 women (12%) in the TMMR group versus 65/226 (29%) in the other groups had a postvoid residual of more than 50 ml (*p* < 0.001; Chi-square test). Length of hospital stay correlated with length of catheter use (*r* = 0.66, *p* < 0.001) and operating time (*r* = 0.28; *p* = 0.002). The time of first postoperative bowel motion was not different between groups (median 4, range 1–8; *p* = 0.265), but more women needed laxatives more than once in the TMMR group (19/35; 54%) compared to LARVH (36/98; 37%), VALRH (34/104; 34%) and VARRH groups (6/24; 25%; *p* < 0.001).

Of the 261 women, 131 women completed the questionnaire (53%; 45 LARVH, 51 VALRH, 10 VARRH, 25 TMMR). Groups did not differ regarding age, BMI and parity (Table 3). The analysis of women who did and those who did not reply did not show a difference in age, BMI, TNM, Piver-Rutledge classification and length of follow-up (data not shown). Given the low number in the robotic group, we combined the laparoscopic and robotic cases as operating steps were similar and analysis of complications and outcomes did not reveal statistically relevant differences apart from operating time (vaginally assisted laparoscopic or robotic radical hysterectomy = VALRRH, *n* = 61). Median follow-up time was significantly shorter in the TMMR group at 16 months (*p* < 0.001; Table 2). One woman completed the questionnaire at 4 months and five women between 6 and 7 months, the others between 12 and 26 months. In the LARVH group, 96% completed the questionnaire more than 24 months after surgery and 69% of the women in the VALRRH group (*p* > 0.05; Table 2).

**Table 2:** Displayed are follow-up time, radiation therapy, pelvic floor questionnaire domain scores and improvement scales as well as postoperative impression of a short vagina in the surgical groups (LARVH-laparoscopically assisted radical vaginal hysterectomy, VALRRH-vaginally assisted laparoscopic or robotic radical hysterectomy, TMMR-laparoscopic total mesometrial resection)

	LARVH *N* = 45	VALRRH *N* = 61	TMMR *N* = 25	*p*
Follow-up time (months; median, range)	70 (5–103)	36 (5–76)	16 (4–26)	< 0.001^a^
Radiation therapy	6 (13%)	13 (21%)	6 (24%)	0.460^b^
Subjective impression Bladder function				0.734^a^
No change Worsened Improved	13 (30%) 31 (70%)	22 (37%) 34 (57%) 4 (7%)	9 (36%) 11 (44%) 5 (20%)	
Subjective impression Bowel function				0.024^a^
No change Worsened Improved	25 (57%) 19 (43%)	30 (50%) 28 (47%) 2 (3%)	6 (24%) 18 (72%) 1 (4%)	
Bladder function score (median, range)	2 (0–5.8)	1.8 (0–6.9)	1.1 (0–6.4)	0.362^a^
Bowel function score (median, range)	1.5 (0.3–5.3)	1.8 (0–7.1)	2.9 (0.9–5.9)	0.001^a^
Prolapse domain score (median, range)	0 (0–4)	0 (0–6.7)	0 (0–0.8)	0.278^a^
Sexual function score (median, range)	1.4 (0–10)	2.4 (0–10)	3.3 (0–6.7)	0.178^a^
Global Pelvic Floor dysfunction score (median, range)	5.4 (0.3–18.9)	5.9 (0.8–19.8)	8 (2–15.4)	0.316^a^
Impression short vagina	11 (32%)	19 (41%)	6 (24%)	0.716^b^

Percentages do not necessarily sum up to 100 due to rounding

^a^Kruskal-Wallis test

^b^Chi-square test

Analysis of the subjective impression of changes (Table 2) showed that 70%, 57% and 44% in the LARVH, VALRRH and TMMR groups, respectively, reported subjective deterioration of bladder function (*p* = 0.734). Stress urinary incontinence was present postoperatively in 81/130 women (62%) and overactive bladder symptoms in 50/131 (38%). Prevalence did not differ between surgical groups. Age, operating technique and Piver-Rutledge class did not impact on bladder function scores although overactive bladder including nocturia correlated with age (*r* = 0.23; *p* = 0.009).

Bowel function worsened in 72% after TMMR which is significantly more often compared with LARVH (43%) and VALRRH (47%; *p*=0.024; Table 2). Women after TMMR also had worse bowel dysfunction scores and reported more frequently constipation, straining at defecation and incomplete bowel emptying, (60 versus 39% and 44%, respectively; *p*=0.004). They also described bowel dysfunction as more bothersome than women in the other groups (*p*=0.001). Age and postoperative radiation therapy were associated with fecal urgency and fecal incontinence for loose stool (*p*<0.021).

The sexual function domain of the questionnaire was completed by 122 women. Of those, 87 (71%) were sexually active at follow-up without differences between surgical groups. Reasons for sexual abstinence were lack of partner (47%), partner impotent (15%), dyspareunia (12%), vaginal dryness (6%) and low desire (6%). Our additional questions were answered by a variable number of women. Worsened ability to climax during sexual activity was reported by 33/99 (33%) women (10/33 after LARVH; 18/49 after VALRRH; 5/17 after TMMR; *p*=0.249). Reduced desire described 39/105 (37%) women, superficial dyspareunia 21/94 (22%), deep dyspareunia 32/94 (34%) and both, introitus and deep pain 10/94 (11%) women without differences between groups. Thirty-eight percent of women (36/96) considered their vagina too short after the operation and 28% (27/96) too narrow or too tight without differences between groups (Table 3). These symptoms were associated with each other (*p*=0.006) and also with postoperative radiation (*p*<0.034). Deep dyspareunia was associated with a short vagina (*p*<0.001). Women with postoperative radiation regardless of surgical technique had a significantly higher (worse) sexual function score (3.3 versus 1.4; *p*<0.001). Coital incontinence was present in 21/93 women (23%). Sexual symptoms were considered moderately and greatly bothersome by 40% which was associated with postoperative radiation (*p*=0.017).

**Table 3:** Comparison of women after laparoscopic/robotic radical hysterectomy or TMMR, controls and urogynaecological patients (LARVH-laparoscopically assisted radical vaginal hysterectomy, VALRH-vaginally assisted laparoscopic radical hysterectomy, TMMR-laparoscopic total mesometrial resection)

	LARVH *N* = 45	VALRRH *N* = 61	TMMR *N* = 25	Controls *N* = 24	Urogyn *N* = 66	p*
Age (years; median, range)	43 (25–73)	45 (27–76)	51 (30–68)	46 (25–67)	51^1^ (30–81)	0.015^a^
Parity (median, range)	1 (0–3)	1 (0–4)	1 (0–4)	1 (0–4)	2 (0–5)	0.654
Bladder function score (median, range)	2 (0–5.8)	1.8 (0–6.9)	1.1^b^ (0–6.4)	0.7^b^ (0–2)	3.2^c^ (0–6.7)	< 0.001^b, c^
Bowel function score (median, range)	1.5 (0.3–5.3)	1.8 (0–7.1)	2.9^d^ (0.9–5.9)	0.9^e^ (0–3.2)	1.8 (0–5.6)	< 0.001^d, e^
Prolapse domain score (median, range)	0 (0–4)	0 (0–6.7)	0 (0–0.8)	0 (0–4)	0.1^f^ (0–7.3)	< 0.001^f^
Sexual function score (median, range)	1.4 (0–10)	2.4 (0–10)	3.3 (0–6.7)	0^g^ (0–3.7)	1.9 (0–5.2)	0.001^g^
Global PF dysfunction score (median, range)	5.4 (0.3–18.9)	5.9 (0.8–19.8)	8 (2–15.4)	2.6 (0–11.4)	9.3 (2.1–15.9)	< 0.001^h^

^*^Kruskal–Wallis tests

^a^post hoc comparison shows urogynaecological patients were significantly older compared to LARVH only

^b^Controls had a significantly lower (better) bladder domain score compared with all groups apart from TMMR

^c^Urogynaecological patients had a significantly higher (worse) bladder domain scores compared with all other groups

^d^TMMR patients had a significantly higher (worse) bowel function score compared with all groups

^e^Controls had a significantly lower (better) bowel domain score

^f^The prolapse symptom score was significantly higher (worse) in the urogynaecological patients compared with all groups

^g^Sexual function scores were significantly lower (better) in the control group

^h^Global PF scores in the RH/TMMR groups were significantly higher (worse) compared to the control group and lower (better) compared with urogynaecological patients

**Fig. 1:**
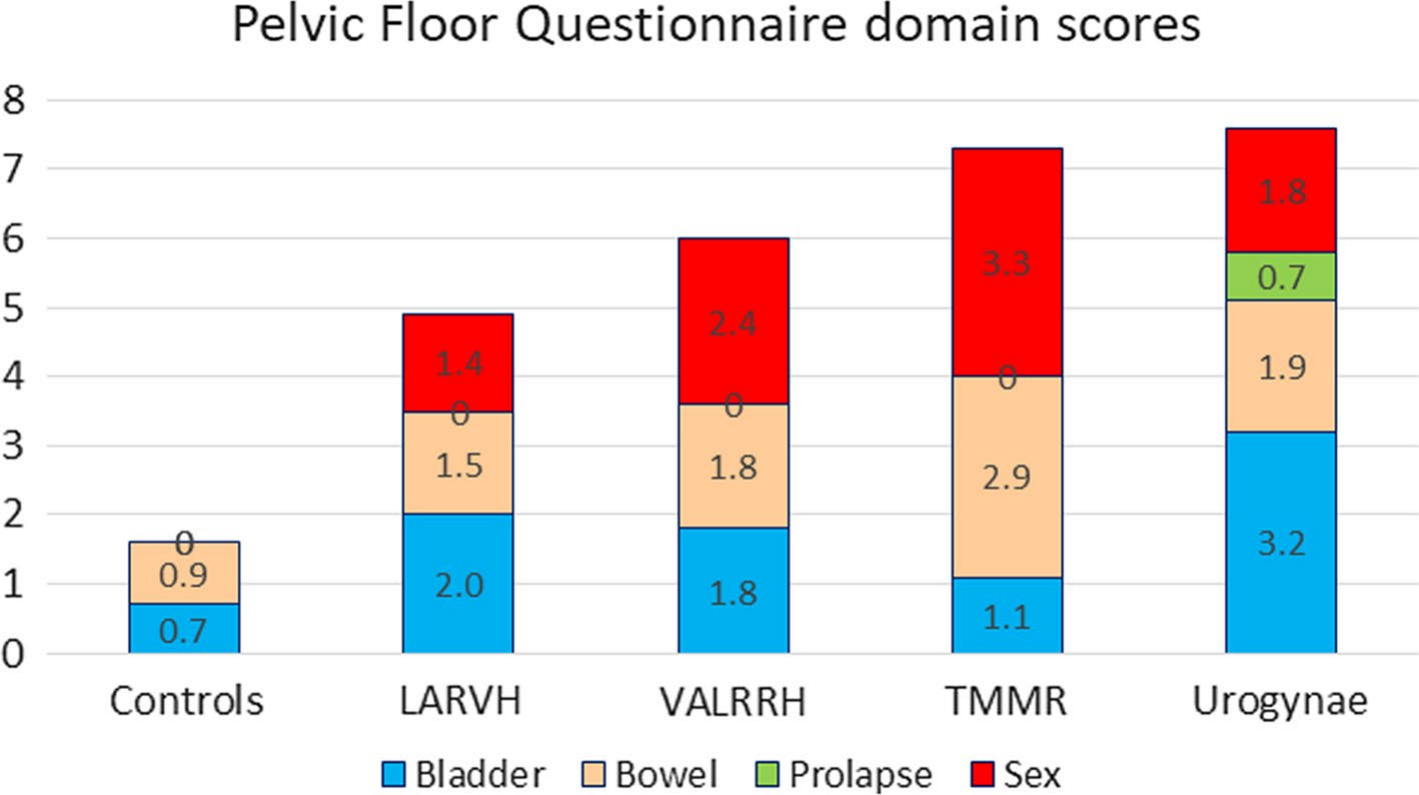
Stacked column plot of median pelvic floor domain scores in the different surgical groups compared to controls and urogynaecological patients. The global pelvic floor questionnaire scores in the surgical groups were significantly higher (worse) compared to the control group and lower (better) compared with urogynaecological patients (LARVH-laparoscopically assisted radical vaginal hysterectomy, VALRH-vaginally assisted laparoscopic radical hysterectomy, TMMR-laparoscopic total mesometrial resection)

Compared to controls, women after surgery showed significantly higher bladder, bowel and sexual dysfunction scores (*p < *0.001, Table 3, Fig. 1). Symptoms of pelvic organ prolapse were not more common than in controls (*p* = 0.720). When LARVH, VALRRH and TMMR groups were separately compared with controls, bladder function was similar to controls only in the TMMR group. Bowel dysfunction was significantly more frequently reported as bothersome after TMMR compared to other surgical groups as well as to urogynecological patients and controls (*p* ≤ 0.004). Urogynecological patients were significantly older than women in the LARVH group, but there were no differences to all other groups (Table 3).

After LARVH, 28/45 women (62%) had clinically relevant self-reported lymphedema, after VALRRH 39/61 (64%) and after TMMR 9/25 (36%; *p* = 0.045; Chi-square test). Recurrent disease at follow-up was present in two (4%), three (5%) and one (4%), respectively (*p* = 0.981).

## Discussion

This study focused on patient-centered outcomes and utilized a validated pelvic floor questionnaire to evaluate the impact of different minimally invasive radical hysterectomy techniques or total mesometrial resection on pelvic floor function. All evaluated surgical techniques resulted in impaired bladder, bowel and sexual function. Pelvic floor dysfunction scores were clinically and statistically significantly worse compared with controls.

Laparoscopic TMMR resulted in early recovery of bladder emptying but led to aggravated bowel dysfunction including constipation and incomplete bowel emptying. The difference in bowel domain scores between TMMR, other surgical groups, urogynecological patients and controls was ≥ 1, which is the estimated minimal important difference of the Australian Pelvic Floor Questionnaire [21]. Interestingly, subjective bothersomeness of bowel dysfunction was greater after TMMR even compared with urogynecological patients. Although bladder dysfunction after TMMR was similar to controls, 44% of women after TMMR reported worsened bladder function. Apart from significantly worse bowel function after TMMR the differences of pelvic floor dysfunction between surgical techniques were small. Surgical radicality and nerve sparing aspects can be considered similar between LARVH and VALRRH, only the TMMR technique differs with Müllerian compartment resection. Although the TMMR technique claims the preservation of nerve structures, the presacral space is dissected which might lead to constipation comparable to complications after rectopexy [22].

We found a high rate of dyspareunia with 67% of all women complaining of introitus and/or deep pain during intercourse. A shortened vagina was reported frequently after surgery and was associated with deep dyspareunia. Radiation therapy aggravated sexual function.

In contrast to our findings, published urinary function data for abdominal TMMR show higher rates of overactive bladder and stress urinary incontinence but less constipation [23]. Whether this is an effect of the laparoscopic technique remains open. A small prospective case series demonstrated a high rate of early postoperative urodynamic detrusor overactivity which persisted long term in 6/9 women after nerve-sparing radical hysterectomy [24]. Given our study design, we might have missed this effect. Compared with our control group as well as published community cohorts, the prevalence of stress and urge urinary incontinence appeared similar [25].

Our data for first postoperative defecation on day 3 or 4 are similar to other studies [8, 26]. Postoperative constipation after radical hysterectomy has been reported in 19%[27]-35% [28], decreasing to 9% after 5 years [29]. This corresponds to our results with exception of the TMMR group, which had significantly more often defecation problems. Given the significantly shorter follow-up time after TMMR, it remains open whether these symptoms recover after 2 years.

A postvoid residual of more than 50 ml was considered pathological in our institution [16]. This cut-off might not be standard in other centers and we might overestimate voiding dysfunction. Hospital stay was rather long between 8–12 days, but is consistent with national practice. It can also be explained with the longer catheter use.

When interpreting our results, it has to be taken into account that pelvic floor dysfunction is very common in women. Urinary incontinence has a high prevalence of more than 50% in postmenopausal women [25]. Data evaluated in this study do not seem particularly high compared with urogynecological patients but well above rates established in non-urogynecological controls. Symptoms were also considered more bothersome than in controls. Our results are in contrast to Novackova et al. [30]. They found less stress urinary incontinence and postoperative urodynamic evaluation did not differ significantly 12 months after nerve-sparing radical hysterectomy. The differences might be attributed to different nerve-sparing techniques but also to their lack of validated questionnaires and short follow-up time [30].

Apart from bowel function after TMMR, PFQ domain scores were lower compared with urogynecological patients. As women in the control groups had not undergone hysterectomy, comparison of pelvic floor symptoms might be considered inappropriate. However, a systematic review of urodynamic outcomes after hysterectomy for benign diseases showed that urinary function is not adversely affected and might improve after hysterectomy [31]. We had purposely chosen urogynecological patients without any pelvic surgery to reduce possible influences as historically many hysterectomies had been performed for pelvic organ prolapse [32].

Regardless of the surgical technique, adjuvant radiation therapy lead to more pelvic floor dysfunction including constipation, excessive straining and incomplete bowel emptying as well as sexual problems. This is in accordance with others who also reported lower general health-related quality of life after radiotherapy [33]. Especially sexual dysfunction was worse after radiotherapy which has been described before [27, 33].

The rate of dyspareunia was high at 67%. A similar rate was reported in women after robotic radical hysterectomy [34]. Given the creation of a vaginal cuff enclosing the cervix prior to radical hysterectomy, the VALRRH groups would be most prone to a shortened vagina. More women in this group (41 versus 32% and 24%) reported this without a statistically significant difference. Although the symptom of a short vagina has been reported before [34–36], our study demonstrated that a subjectively short and narrow vagina interferes with sexual intercourse. Höckel et al. reported only few problems after abdominal TMMR but there was no systematic analysis of sexual aspects [23]. Sowa et al. compared TMMR with the classic Wertheim-Meigs operation and did not find differences regarding sexuality [37]. Some studies imply that sexual problems increase with length of follow-up [27]. Long-term evaluation should be considered in all patients after radical hysterectomy due to the fact that survival rates are high and morbidity data after 5 years are scarce.

Postoperative lymphedema is common [7] and our rates are similar to reports after robotic radical hysterectomy [34]. The rate of postoperative lymphedema was lowest after TMMR which also corresponds to the fact that less lymph nodes were dissected (Table 1). We did not assess how much lymphedema interferes with quality of life but others described high levels of distress [7, 34]

### Strengths and weaknesses

Limitations of this study include lack of preoperative data including pelvic floor dysfunction and different lengths of follow-up. This study was not prospectively planned, not randomized and institutional surgical techniques had been transformed according to complication profiles. Furthermore, the Australian Pelvic Floor Questionnaire and its German version has been validated in community-dwelling women [38], patients attending gynecological and urogynaecologic clinics [20] as well as in pregnant and postpartum women [39] but not specifically in gynecological cancer patients. Nevertheless, it is a strength of this study that a validated self-administered instrument including postoperative scales of impression of improvement was used and the minimal important difference is known. Also, the comparison to healthy controls and urogynecological patients provide information on the magnitude of the reported pelvic floor symptoms. In our view, it helps to interpret the severity of pelvic floor symptoms.

Further strengths include that the calculated sample size was reached and the study is powered to evaluate subjective pelvic floor function as a patient-centered outcome. Due to the methodological limitations, effect of postoperative radiotherapy cannot be analyzed in detail. Furthermore, standardization of nerve-sparing techniques are ongoing [40].

## Conclusions

Despite the limitations of this retrospective study, we believe we provided relevant information on patient-centered outcomes of pelvic floor function after different techniques of minimally invasive radical hysterectomy and TMMR to improve counseling of women with cervical cancer. All evaluated minimally invasive techniques for patients with early-stage cervical cancer had a negative impact on pelvic floor function exceeding symptom scores in controls. Laparoscopic TMMR was initially better for bladder function but not long term and resulted in a persisting and significantly higher rate of bowel dysfunction. When choosing an operation for cervical cancer, pelvic floor symptoms should be taken into account and should be part of preoperative counseling, provided that oncologic safety can be assured. For women with existing constipation, e.g., TMMR with a higher rate of postoperative bowel dysfunction might be less appropriate. Preoperative and postoperative assessment of pelvic floor function including sexuality should be mandatory in patients undergoing surgery for cervical cancer.

Raising awareness of possible deterioration of pelvic floor function would enable health care providers to offer better support and early therapy. Future studies on cervical cancer surgery should include validated outcome measures to assess pelvic floor function and especially sexual function. Also, possible prevention strategies should be addressed prospectively.

## Data Availability

Data will be made available on reasonable request.
